# Implantable Electroceutical Approach Improves Myelination by Restoring Membrane Integrity in a Mouse Model of Peripheral Demyelinating Neuropathy

**DOI:** 10.1002/advs.202201358

**Published:** 2022-08-17

**Authors:** Aseer Intisar, Hyun Young Shin, Woon‐Hae Kim, Hyun Gyu Kang, Min Young Kim, Yu Seon Kim, Youngjun Cho, Yun Jeoung Mo, Heejin Lim, Sanghoon Lee, Q. Richard Lu, Yun‐Il Lee, Minseok S. Kim

**Affiliations:** ^1^ Department of New Biology DGIST Daegu 42988 Republic of Korea; ^2^ CTCELLS Corp. Daegu 42988 Republic of Korea; ^3^ SBCure Corp. Daegu 43017 Republic of Korea; ^4^ Well Aging Research Center DGIST Daegu 42988 Republic of Korea; ^5^ Department of Robotics and Mechatronics Engineering DGIST Daegu 42988 Republic of Korea; ^6^ Department of Pediatrics Cincinnati Children's Hospital Medical Center Cincinnati OH 45229 USA; ^7^ Translational Responsive Medicine Center (TRMC) DGIST Daegu 42988 Republic of Korea; ^8^ New Biology Research Center (NBRC) DGIST Daegu 42988 Republic of Korea

**Keywords:** Charcot‐Marie‐Tooth disease, cholesterol, electroceuticals, myelination, myelin membrane integrity, peripheral demyelinating neuropathies, PMP22

## Abstract

Although many efforts are undertaken to treat peripheral demyelinating neuropathies based on biochemical interventions, unfortunately, there is no approved treatment yet. Furthermore, previous studies have not shown improvement of the myelin membrane at the biomolecular level. Here, an electroceutical treatment is introduced as a biophysical intervention to treat Charcot‐Marie‐Tooth (CMT) disease—the most prevalent peripheral demyelinating neuropathy worldwide—using a mouse model. The specific electrical stimulation (ES) condition (50 mV mm^−1^, 20 Hz, 1 h) for optimal myelination is found via an in vitro ES screening system, and its promyelinating effect is validated with ex vivo dorsal root ganglion model. Biomolecular investigation via time‐of‐flight secondary ion mass spectrometry shows that ES ameliorates distribution abnormalities of peripheral myelin protein 22 and cholesterol in the myelin membrane, revealing the restoration of myelin membrane integrity. ES intervention in vivo via flexible implantable electrodes shows not only gradual rehabilitation of mouse behavioral phenotypes (balance and endurance), but also restored myelin thickness, compactness, and membrane integrity. This study demonstrates, for the first time, that an electroceutical approach with the optimal ES condition has the potential to treat CMT disease and restore impaired myelin membrane integrity, shifting the paradigm toward practical interventions for peripheral demyelinating neuropathies.

## Graphical abstract

An electroceutical approach to improve myelination in a mouse model of Charcot‐Marie‐Tooth disease is presented. Following an optimal electrical stimulation (ES) condition, myelination enhancement is confirmed at in vitro, ex vivo, and in vivo stages. Biomolecular analysis shows restored myelin membrane integrity via improvements in PMP22 and cholesterol distribution. Implantable ES to the mouse promotes gradual rehabilitation of behavioral phenotypes.

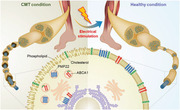

## 1 Introduction

The myelin sheath serves several crucial roles for the proper function of the nervous system: it helps maintain saltatory conduction during neuronal transmission, provides beneficial trophic factors to neurons, and aids neuroregeneration after nerve injury. Therefore, the loss of myelin in the peripheral nervous system (PNS) causes peripheral demyelinating neuropathies, such as Charcot‐Marie‐Tooth (CMT) disease, the most prevalent inherited peripheral neuropathy worldwide.^[^ ^1^ ^]^ Its symptoms, including skeletal muscle atrophy, hand and foot deformities, gait abnormalities, and numbness in limbs, present substantial impediments to the patients’ quality of life. It has two possible pathophysiologies, demyelination or axonal degeneration in the PNS, with the former being more prevalent.^[^ ^2^ ^]^ Although many pharmacological interventions have been proposed, unfortunately, an established treatment for CMT disease remains lacking.^[^ ^3^ ^]^ For example, ascorbic acid‐based treatment has been suggested, but it failed to demonstrate significant benefits during the clinical trial.^[^ ^4^ ^]^ Other pharmacological interventions include exogenous phosphatidylcholine supplementation and increasing serum cholesterol level via a high‐fat diet, which were shown to ameliorate demyelination in mouse models.^[^ ^5^ ^]^ However, since elevated phosphatidylcholine intake and high serum cholesterol levels are both risk factors for cardiovascular diseases, the strategies might be infeasible in clinical translation.^[^ ^6^ ^]^ Alternatively, gene therapy has been attempted to treat CMT disease in mouse models;^[^ ^7^ ^]^ however, there are ethical concerns and regulatory barriers in human application. Given that the biochemical interventions have not been successful, a novel therapeutic approach is urgently required, making it compelling to pursue a biophysical intervention.

Electric signals are the communication language of the nervous system. Hence, electroceuticals are emerging as effective therapeutics toward neuropathies.^[^ ^8^ ^]^ For example, in the central nervous system (CNS), electroceuticals are already used as interventions for Parkinson's disease, epilepsy, and spinal cord injury.^[^ ^9^ ^]^ Recently, electroceuticals have also shown promise toward obsessive‐compulsive behavior and treatment‐resistant depression.^[^ ^10^ ^]^ Electrical stimulation (ES) has also shown positive effects in the PNS. ES promotes neurite outgrowth and the release of neurotrophic factors from peripheral neurons,^[^ ^11^ ^]^ enhances Schwann cell (SC) migration,^[^ ^12^ ^]^ and also improves nerve regeneration in vivo following peripheral nerve injury.^[^ ^13^ ^]^ Recently, we have also shown that ES enhances myelination in the mouse dorsal root ganglion (DRG).^[^ ^14^ ^]^ Although electroceuticals have shown promising results in acquired peripheral neuropathies, which can regenerate to some extent without external therapy, their potential to treat inherited peripheral neuropathies, such as CMT disease, remains unexplored.

One of the most widely utilized animal models for demyelinating CMT disease is the Trembler‐J (Tr‐J) mouse, which exhibits gradual demyelination in the PNS and a decline in physical performance, resembling the disease progression in human demyelinating CMT patients.^[^ ^15^ ^]^ Similar to the majority of demyelinating CMT patients and animal models, Tr‐J mouse carries a mutation in peripheral myelin protein 22 (PMP22), which leads to the retention of PMP22 in the perinuclear region and a dispersed distribution in the myelin membrane.^[^ ^16^ ^]^ Due to the close association between PMP22 and the cholesterol transporter ATP binding cassette subfamily A member 1 (ABCA1), the Tr‐J myelin membrane also shows a dispersed distribution of cholesterol, one of the most abundant and essential structural lipids of myelin.^[^ ^17^ ^]^ The abnormalities in PMP22 and cholesterol distribution compromise the myelin membrane integrity. Therefore, it is important to evaluate the efficacy of a treatment to ameliorate CMT phenotype in the Tr‐J mouse based on the following key parameters: 1) PNS myelination, 2) PMP22 and cholesterol distribution in the myelin membrane, and 3) behavioral phenotype.

**Figure 1:**
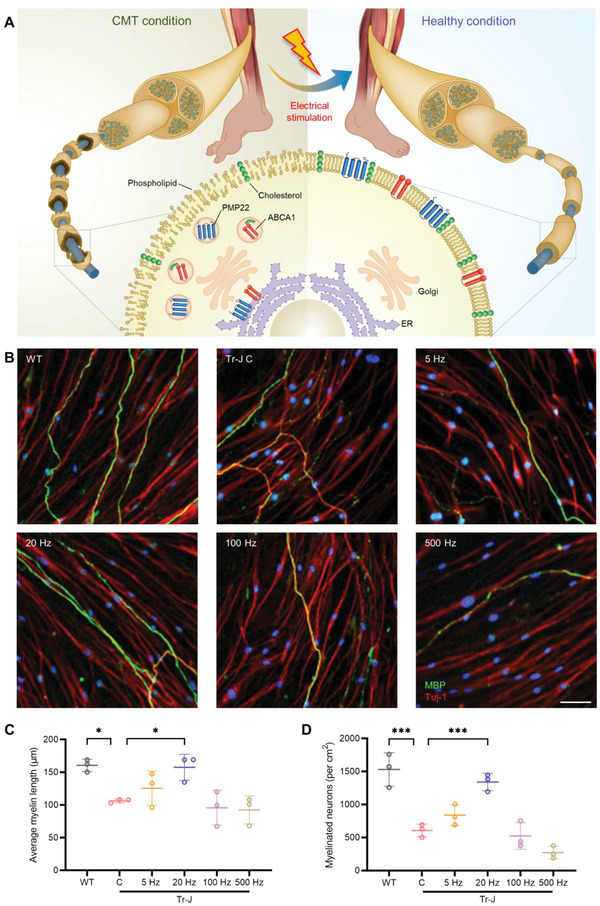
Schematic diagram of the treatment approach and the results of in vitro screening. A) Schematic showing the novel approach to improve myelination in CMT disease via ES and the biomolecular changes that precede the improvement in myelination after ES. ES ameliorates the abnormalities associated with aggregated peripheral myelin protein 22 (PMP22) and ATP binding cassette subfamily A member 1 (ABCA1) near the ER, as well as dispersed cholesterol distribution in the CMT myelin membrane. B) Representative immunostained images of WT and Tr‐J neuron‐SC cocultures at 35 DIV, with the latter treated with ES at 5, 20, 100, and 500 Hz. Neurons were stained with beta III tubulin (Tuj‐1) and SCs were stained with MBP. Scale bar, 50 µm. C) Average myelin length (*n* = 3) in the control WT and Tr‐J cocultures, and Tr‐J cocultures treated with ES at different frequencies. D) Density of myelinated neurons (*n* = 3) in the control WT and Tr‐J cocultures, and Tr‐J cocultures treated with ES at different frequencies. The average myelin length was measured from the MBP (green) channel from 10× images using the NeuronJ plugin in ImageJ. Images from three biologically distinct samples per condition were used, and the average of ten myelin segments per image was taken for each sample. Myelinated neurons per cm^2^ were measured using the colocalization of Tuj‐1 (red) and MBP (green) channels from 10× images using ImageJ, and images from three biologically distinct samples per condition were used. The data are expressed as the mean ± s.d. *p < 0.05, **p < 0.005, ***p < 0.0005.

Here, we introduce an electroceutical approach to treat the demyelinating CMT disease (**Figure** **1**A). A screening step using in vitro neuron‐SC coculture identified that ES at 50 mV mm^−1^, 20 Hz for 1 h was optimum for improving myelination. The effectiveness of this condition was further verified using the DRG explant, an ex vivo model of the PNS. Biomolecular investigation revealed that ES reduced PMP22 retention in the perinuclear region and improved distribution along the Tr‐J myelin membrane. The imaging of myelin lipids via time‐of‐flight secondary ion mass spectrometry (ToF‐SIMS) showed that ES mitigated the dispersed cholesterol distribution in the Tr‐J myelin. Thus, the amelioration of PMP22 and cholesterol distribution abnormalities restored the integrity of the myelin membrane. Lastly, flexible cuff electrodes were implanted onto the sciatic nerves of Tr‐J mice, and the optimal ES was provided, showing gradual rehabilitation of the behavioral phenotype of Tr‐J mice. After ES, sciatic nerve analysis showed not only increased myelin thickness and compactness but restored myelin membrane integrity. The effectiveness of ES was demonstrated systematically, including in vitro screening for optimal ES condition, reconfirmation of the optimal condition with ex vivo model, biomolecular investigation, as well as in vivo behavioral and histopathological analysis.

## 2 Results

### 2.1 Specific ES Promotes Myelination in Tr‐J Neuron‐SC Coculture

As no study has yet reported the efficacy of ES in ameliorating demyelination in inherited PNS neuropathies, we first screened for the ES condition that would optimally improve myelination in an in vitro coculture of neurons and SCs from Tr‐J mice.^[^ ^18^ ^]^ The methodological contents regarding the neuron‐SC extraction and coculture are described in the Experimental Section, and shown in Figure S1, Supporting Information. The custom‐made ES platform is shown in Figure S2, Supporting Information. Since electric fields stronger than 50 mV mm^−1^ can deteriorate the morphology of SCs,^[^ ^19^ ^]^ ES was applied in the 5–500 Hz range at 50 mV mm^−1^ for 1 h. Myelination was evaluated via immunostaining after 4 weeks of coculture (Figure 1B), using myelin basic protein (MBP) as the SC marker and beta III tubulin (Tuj‐1) as the neuron marker. Quantification was carried out for the average myelin length and the density of myelinated neurons, as they are determinant factors for PNS axonal functionality.^[^ ^20^ ^]^

Compared with cocultures derived from wild‐type (WT) mice, those derived from Tr‐J mice (control) had 34% shorter myelin segments (Figure 1C) and 60% lower density of myelinated neurons (Figure 1D). Following application of 20 Hz ES, there was a significant improvement in these parameters, leading to 49% longer myelin segments and 121% increase in the density of myelinated neurons compared with the cocultures derived from the Tr‐J control. In contrast, use of a relatively higher frequency of 500 Hz led to slightly shorter myelin segments (13%) and a markedly lower density of myelinated neurons (55%) compared with the Tr‐J control; moreover, myelin marker expression was dispersed and faint, suggesting poor myelin quality. These results could be comprehended with regards to previous PNS studies: while relatively low frequencies (1–20 Hz) have been shown to have a beneficial effect on neurotrophic factor release, axonal regeneration, and neurite outgrowth,^[^ ^11^ ^]^ relatively higher frequencies, such as 200 Hz, led to the exhaustion of action potential (AP) in PNS neurons.^[^ ^21^ ^]^

The screening results were confirmed by comparing the expression of genes encoding MBP and c‐Jun via quantitative reverse transcription polymerase chain reaction (RT‐qPCR) (Figure S3A,B, Supporting Information). MBP is a crucial component of compact myelin,^[^ ^22^ ^]^ while c‐Jun negatively regulates PNS myelination by inducing SC de‐differentiation.^[^ ^23^ ^]^ The RT‐qPCR data showed that MBP expression was significantly downregulated (66%) and c‐Jun expression was significantly upregulated (259%) in the Tr‐J control compared to the WT, which was consistent with previous findings.^[^ ^5b^ ^,1^ ^5c^ ^]^ These data also showed that, compared to the Tr‐J control, the greatest extent of upregulation in MBP expression (98%) and downregulation in c‐Jun expression (45%) resulted from 20 Hz ES. According to these results, we found that ES at 20 Hz and 50 mV mm^−1^ for 1 h was optimal to improve myelination in the Tr‐J in vitro.

### 2.2 Verification of the ES Effect on Myelination in the Tr‐J Ex Vivo Model

**Figure 2:**
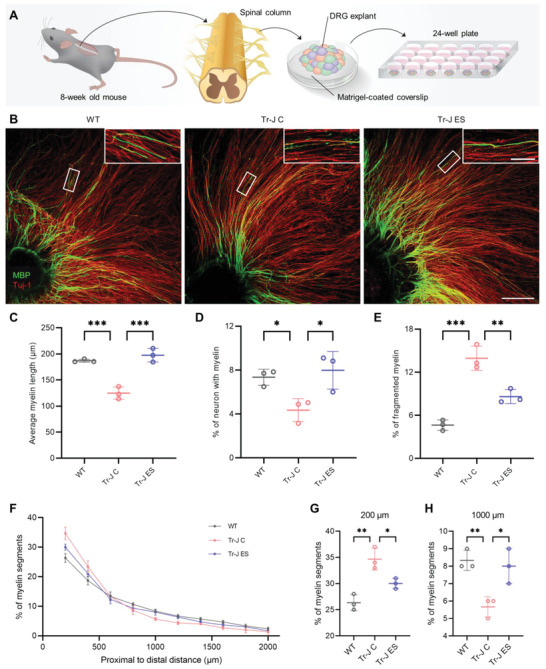
ES ameliorates demyelination in Tr‐J ex vivo. A) Schematic illustration of DRG explant extraction from the spinal column of an adult mouse and the subsequent culture. B) Representative immunostained images of WT, Tr‐J control, and Tr‐J ES DRGs. The image in the inset shows the enlarged region marked in the respective images. Scale bars, 500 µm (main) and 100 µm (inset). C) Average myelin length, D) percentage of myelinated neurons, and E) percentage of fragmented myelin segments in WT, Tr‐J control, and Tr‐J ES DRGs (*n* = 3). F) Percentage of myelin segments in the DRG in each 200 µm radial slice, starting from the edge of the explant (*n* = 3). G) Percentage of myelin segments in a slice 200 µm from the edge of the explant (*n* = 3). H) Percentage of myelin segments in a slice 1000 µm from the edge of the explant (*n* = 3). The average myelin length was measured from the MBP (green) channel from 4× images using the NeuronJ plugin in ImageJ. Images from three biologically distinct samples per condition were used, and the average of ten myelin segments per image was taken for each sample. Myelinated neurons per cm^2^ were measured using the colocalization of Tuj‐1 (red) and MBP (green) channels from 4× images using ImageJ, and images from three biologically distinct samples per condition were used. The percentage of fragmented myelin and percentage of myelin in 200 µm slices were measured using the MBP (green) channel from the 4× images of three biologically distinct samples per condition. The data are expressed as the mean ± s.d. *p < 0.05, **p < 0.005, ***p < 0.0005.

In order to further verify the improvement in myelination with ES, it is necessary to utilize a model that more closely mimics the PNS environment, such as the DRG explant. The DRG explant is an ex vivo PNS model that preserves the native cell population, cell–cell interactions, extracellular matrix, and biochemical cues. As such, DRG explants have been utilized in studies of neuroplasticity, myelination, and drug discovery for neuropathies in the PNS.^[^ ^24^ ^]^ Here, the DRG explant was used as an ex vivo PNS model to verify the myelination improvement with the optimal parameters determined from the in vitro screening (20 Hz and 50 mV mm^−1^ for 1 h). The methodological contents regarding the DRG extraction and culture are described in the Experimental Section, and shown in **Figure** **2**A and Figure S4, Supporting Information.

ES significantly enhanced the myelination of the DRG explants of Tr‐J mice relative to that of Tr‐J control, as demonstrated by immunostaining after 5 weeks of total culture (Figure 2B and Figure S4C, Supporting Information). The DRGs from the Tr‐J mice contained 34% shorter myelin segments on average (Figure 2C) and 41% fewer myelinated neurons (Figure 2D), compared to the WT DRGs. ES led to significant improvements in both of these parameters—58% longer myelin segments (Figure 2C) and 83% more myelinated neurons (Figure 2D)—bringing them closer to the levels observed in WT DRGs. Inspection of the myelin quality in the DRGs revealed that, while the WT DRGs showed myelin segments with good uniformity, the Tr‐J DRGs exhibited almost threefold higher fragmented myelin segments (13.9% in Tr‐J C and 4.6% in WT) where the neurons were exposed due to sparse myelin, indicating the poor quality of myelin (Figure 2E). ES treatment ameliorated this phenomenon, reducing the percentage of fragmented myelin by 38% compared to the control Tr‐J DRGs. Therefore, we found that ES improved the extent of myelination and reduced myelin fragmentation in Tr‐J DRG explants.

The demyelination in CMT usually becomes more severe in the distal region of the axon compared to the proximal, making the symptoms, such as muscle atrophy and loss of neuronal function, exacerbated in the distal region of the limb.^[^ ^25^ ^]^ For this reason, an optimal treatment for CMT should ameliorate demyelination comparatively more in the distal region than in the proximal. Therefore, the effectiveness of ES toward ameliorating demyelination in both the proximal and distal regions of the axon was evaluated. This was done by counting the number of myelin segments in 200 µm regions with increasing proximal to distal distance from the center of the DRG. The results showed that, in every 200 µm region, while the Tr‐J control DRGs had fewer myelin segments compared with the WT, ES led to an increase in the number of myelin segments in the Tr‐J DRGs (Figure S4D, Supporting Information). If the extent of myelination improvement in the distal region is sufficiently dominant relative to the proximal, it might alter the distribution of myelin in the DRGs. To investigate this, the percentage of myelin segments in each 200 µm region was calculated, based on the total number of myelin segments in the whole DRG. Compared with WT, the distribution of myelin in the Tr‐J control DRGs was more skewed toward the proximal region, indicating more severe demyelination in the distal region. After ES, the distribution of myelin in the Tr‐J DRGs was restored toward the WT (Figure 2F). Compared with the WT DRGs, the Tr‐J control DRGs had a significantly higher percentage of myelin segments in a proximal region (Figure 2G) and a significantly lower percentage of myelin segments in a distal region (Figure 2H). In contrast, compared with the Tr‐J control DRGs, the Tr‐J ES DRGs had a significantly lower percentage of myelin segments in the proximal region (Figure 2G) and a significantly higher percentage of myelin segments in the distal region (Figure 2H). These results suggested that ES treatment was effective in ameliorating the exacerbated distal myelination observed in CMT.

### 2.3 ES Promotes PMP22 and ABCA1 Distribution along the Myelin Membrane

In addition to the improvements in myelination, we sought to examine whether ES improved the defects in protein distribution in Tr‐J. The Tr‐J mouse has a point mutation in the gene encoding PMP22,^[^ ^16b^ ^]^ a transmembrane protein that plays an essential role in the architecture of the myelin membrane and the formation of lipid rafts.^[^ ^26^ ^]^ Although healthy PMP22 undergoes posttranslational modification in the endoplasmic reticulum (ER) before being translocated to the membrane,^[^ ^27^ ^]^ it has been well reported that mutated PMP22 is retained near the ER and the perinuclear region of SCs in demyelinating CMT disease,^[^ ^28^ ^]^ potentially arising from the misfolding of the mutant forms of PMP22.^[^ ^29^ ^]^ In addition, ABCA1 plays a crucial role in shuttling cholesterol—one of the key lipid components of the myelin membrane—between the ER and the plasma membrane.^[^ ^17^, ^30^ ^]^ Due to the close association of ABCA1 and PMP22 within SCs,^[^ ^17c^ ^]^ Tr‐J also exhibits impairment in the cholesterol distribution in the myelin membrane.^[^ ^17b^ ^]^

**Figure 3:**
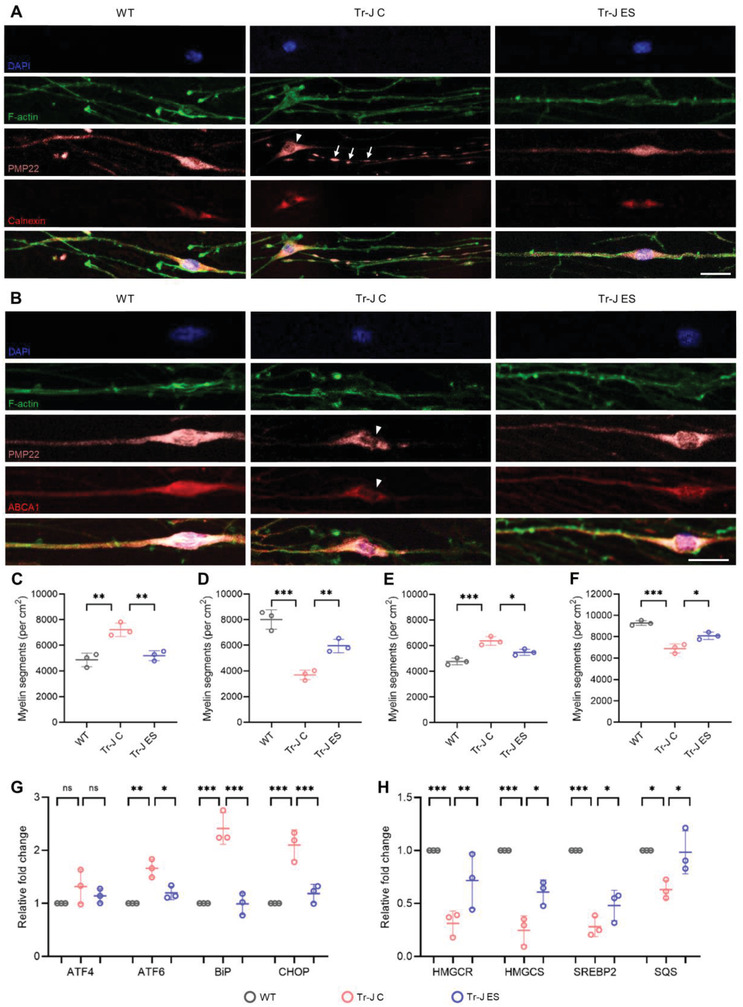
Amelioration of abnormalities in PMP22/ABCA1 distribution, ER stress, and cholesterol biosynthesis in Tr‐J after ES. A) Representative immunostained images of the colocalization of PMP22 with ER (calnexin used as marker) in the myelin of WT, Tr‐J control, and Tr‐J ES cocultures. The arrowhead shows the PMP22 retention around the ER, and the arrows show the intermittent PMP22 distribution along the myelin. Scale bar, 25 µm. B) Representative immunostained images of the colocalization of PMP22 with ABCA1 in the myelin of WT, Tr‐J control, and Tr‐J ES cocultures. The arrowheads show the PMP22 and ABCA1 colocalization and retention around the ER. Scale bar, 25 µm. C) Density of myelin segments with PMP22 restricted around the ER (*n* = 3). D) Density of myelin segments with uniform PMP22 distribution (*n* = 3). E) Density of myelin segments with ABCA1 restricted around the ER (*n* = 3). F) Density of myelin segments with uniform ABCA1 distribution (*n* = 3). Quantitative reverse transcription polymerase chain reaction (RT‐qPCR) analysis for gene expression related to G) ER stress and H) cholesterol biosynthesis (*n* = 3). Quantification of PMP22 and ABCA1 distribution was performed using individual ABCA1 (red) and PMP22 (pink) channels from 10× images using ImageJ and using 4′,6‐diamidino‐2‐phenylindole (blue) as a reference to identify the perinuclear region. In each quantification, images from three biologically distinct samples per condition were used. The data are expressed as the mean ± s.d. *p < 0.05, **p < 0.005, ***p < 0.0005.

Regarding the abnormality in the perinuclear retention of PMP22 and ABCA1, we examined the colocalization of PMP22 with respect to the ER and ABCA1 in the myelin of the neuron‐SC coculture. While the WT coculture showed uniform PMP22 distribution along the myelin, the PMP22 in the Tr‐J coculture was mostly retained around the ER and intermittently distributed along the myelin (**Figure** **3**A). Compared with the WT coculture, the Tr‐J coculture showed a 48% higher density of myelin segments where PMP22 was retained near the ER (Figure 3C), and a 54% lower density of myelin segments where PMP22 was distributed uniformly along the myelin (Figure 3D). Interestingly, ES treatment produced a clear improvement in this abnormality, resulting in a 28% decline in the density of myelin segments where PMP22 was retained near the ER and a 61% improvement in the density of myelin segments where PMP22 was distributed uniformly along the myelin. The distribution of ABCA1 was similar to that of PMP22, affirming the close association of the two proteins (Figure 3B). Compared with the WT coculture, the Tr‐J coculture showed a 33% higher density of myelin segments with ABCA1 expression near the ER (Figure 3E) and a 26% lower density of myelin segments with ABCA1 distributed uniformly along the myelin (Figure 3F). ES treatment resulted in a 14% decline in the density of myelin segments where ABCA1 was retained near the ER and a 17% improvement in the density of myelin segments with ABCA1 expression distributed uniformly along the myelin. The data provide mechanistic insight toward the improvement in Tr‐J myelination after ES treatment, which was potentially brought about via the amelioration of the PMP22/ABCA1 distribution abnormality observed in Tr‐J myelin.

Since the misfolding of PMP22 can elevate ER stress in demyelinating CMT and the reduced ER stress via curcumin treatment previously showed beneficial effects in improving myelination in CMT,^[^ ^31^ ^]^ we investigated whether the improvement in PMP22 distribution was accompanied by a reduction in ER stress. We measured the expression of representative ER stress genes—ATF4 (activating transcription factor 4), ATF6 (activating transcription factor 6), BiP (binding immunoglobulin protein or heat shock 70 kDa protein 5), and CHOP (C/EBP homologous protein or DNA damage‐inducible transcript 3)—in the neuron‐SC coculture one day after ES treatment. While ATF6, BiP, and CHOP were significantly higher expressed in the Tr‐J coculture than in the WT coculture, the expression of ATF6, BiP, and CHOP in the Tr‐J ES coculture was significantly lower than that in the Tr‐J control coculture, the extent of which almost recovered to the levels seen in the WT coculture (Figure 3G). These results indicated that ES treatment improved the PMP22 distribution in Tr‐J myelin and reduced ER stress in Tr‐J neuron‐SC coculture.

### 2.4 Genes Involved in Cholesterol Biosynthesis are Upregulated via ES

The myelin membrane is exceptionally enriched in lipids, with cholesterol being one of the most abundant structural lipids, shown to be essential for myelin membrane synthesis.^[^ ^32^ ^]^ The downregulation of the expression of genes involved in cholesterol biosynthesis leads to demyelination in the PNS.^[^ ^33^ ^]^ Along with an abnormality in PMP22 distribution in the myelin membrane, demyelinating CMT animal models exhibit a deficiency in cholesterol biosynthesis.^[^ ^5^, ^17^ ^]^ Therefore, after applying ES, we investigated the changes in the expression of genes involved in cholesterol biosynthesis—HMGCR (HMG‐CoA reductase), HMGCS (HMG‐CoA synthase), SREBP2 (sterol regulatory element‐binding protein 2), and SQS (squalene synthase)—in the neuron‐SC coculture (Figure 3H). Compared with the WT coculture, while the Tr‐J coculture exhibited significantly lower expression of all four genes, they were significantly upregulated after ES. Notably, the expression level of SQS,^[^ ^34^ ^]^ an essential enzyme for cholesterol biosynthesis—as it catalyzes the reaction that commits the pathway to synthesize cholesterol—was improved to an extent comparable to WT levels. This result suggests that ES improved de novo cholesterol biosynthesis, which is characteristically deficient in demyelinating CMT. The improved de novo cholesterol biosynthesis indicates that exogenous supplementation might not be required for improving myelination in CMT.

### 2.5 ES Restores Myelin Membrane Integrity by Improving Cholesterol Distribution

**Figure 4:**
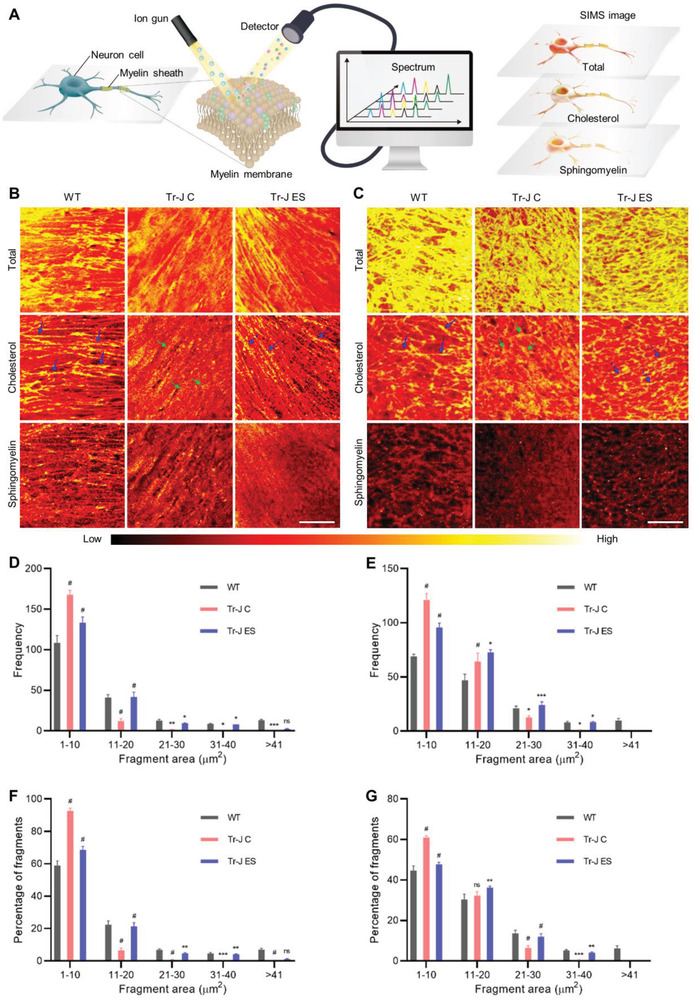
Imaging myelin lipids via time‐of‐flight secondary ion mass spectrometry (ToF‐SIMS) and size distribution of cholesterol‐rich fragments. A) A schematic summarizing the process of ToF‐SIMS imaging. Representative SIMS images of total lipids, cholesterol, and sphingomyelin in the WT, Tr‐J control, and Tr‐J ES samples from B) in vitro coculture and C) ex vivo DRG explant. Scale bars, 100 µm. D) Frequency and F) percentage of cholesterol‐rich fragments of different size groups in in vitro coculture (*n* = 3). E) Frequency and G) percentage of cholesterol‐rich fragments of different size groups in ex vivo DRG explants (*n* = 3). Statistical comparison (two‐way ANOVA) was performed between the following groups: WT versus Tr‐J C and Tr‐J C versus Tr‐J ES. For each condition, images from three biologically distinct samples were used. The data are expressed as the mean ± s.d. *p < 0.05, **p < 0.005, ***p < 0.0005, #*p* < 0.0001.

To further validate whether the improvements in the distribution of PMP22 and ABCA1, as well as the upregulation of genes related to cholesterol biosynthesis resulted in better cholesterol distribution in the myelin membrane, we decided to image the lipids in the myelin membrane. Conventional lipid imaging techniques (such as Filipin staining for cholesterol) are incompatible for imaging membrane lipids, as they are prone to interference from intracellular lipids. To overcome this issue, we analyzed both the in vitro coculture and ex vivo DRG explant models via ToF‐SIMS. In this imaging technique, a beam of positively charged heavy ions hits the cell membrane, and creates fragment molecules by breaking down the lipid molecules. These fragment molecules then pass through a time‐of‐flight analyzer, and a spectrum is derived based on the *m*/*z* ratio and the population of each fragment. An image is then constructed from the spectrum, where the color indicates the density of the lipid molecule present in the region of interest (**Figure** **4**A). Therefore, this state‐of‐the‐art analytical technique is well suited for the bioimaging of lipids in cellular membranes with its molecular level resolution, and the shallow sampling depth enables the imaging of membrane lipids with negligible interference from intracellular lipids.^[^ ^35^ ^]^

In the ToF‐SIMS images, the regions with relatively higher density for the lipid of interest are indicated in yellow. The ToF‐SIMS images of WT in vitro coculture showed several cholesterol‐rich segments, generating solid lines (see the blue arrows in Figure 4B). In contrast, the images from the Tr‐J control coculture showed noticeably fewer cholesterol‐rich segments (see the green arrows in Figure 4B). Whereas the cholesterol‐rich segments in WT were linear and contiguous, the cholesterol‐rich segments in the Tr‐J control were fragmented, indicating cholesterol distribution abnormality in the myelin membrane of the Tr‐J control.^[^ ^17b^ ^]^ Upon ES treatment, the cholesterol‐rich segments in the Tr‐J coculture became noticeably less fragmented (see the blue dotted arrows in Figure 4B), suggesting that ES led to an amelioration of the cholesterol distribution abnormality. Sphingomyelin is another abundant lipid component in the myelin membrane; nevertheless, abnormalities in the distribution of sphingomyelin in Tr‐J myelin have not been reported. Therefore, not unexpectedly, the ToF‐SIMS data exhibited negligible changes in the sphingomyelin distribution relative to those of cholesterol. The observations on the distributions of cholesterol and sphingomyelin in the in vitro coculture model were consistent with those of the ex vivo DRG explant (Figure 4C).

For further analysis, we quantified the distribution of the size of the cholesterol‐rich fragments from ToF‐SIMS images in both the in vitro (Figure 4D,F) and ex vivo (Figure 4E,G) cases, comparing the frequency and percentage of fragments in different size groups. Compared with those in the WT mice, the frequency and percentage of fragments in the smallest size group (1–10 µm^2^) were dominant, and the frequencies and percentages of fragments in medium‐sized groups (21–40 µm^2^) were significantly lower in the Tr‐J mice. This suggests that the Tr‐J myelin membrane shows a more aberrant cholesterol distribution than the WT myelin membrane. Meanwhile, ES significantly decreased the frequency and percentage of fragments in the smallest size group (1–10 µm^2^) and significantly increased the frequencies and percentages of fragments in the medium‐sized groups (21–40 µm^2^). In both the in vitro and ex vivo cases, however, the effect of the application of ES was negligible in the largest fragment group (>41 µm^2^). Based on these results, we found that ES mitigated the dispersed cholesterol distribution in Tr‐J myelin, and in combination with the improvement in PMP22 distribution, this restored the myelin membrane integrity.

### 2.6 Implantable ES Promotes the Rehabilitation of Behavioral Phenotypes of Tr‐J Mice

**Figure 5:**
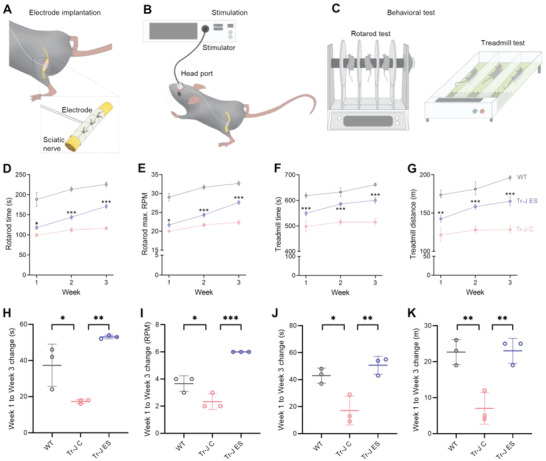
ES improves the behavioral phenotypes of Tr‐J mice. A) Implantation of the cuff electrode onto the sciatic nerve of a Tr‐J mouse. The connecting wires are routed via the subcutaneous layer, and a head port is secured onto the scalp. B) ES is provided on the first day of the week, 30 min per day for 3 consecutive weeks, via a head port connected to a stimulator. C) At the end of each week, rotarod and treadmill tests are conducted to evaluate the physical balance and running endurance, respectively. The maximum D) time and E) RPM achieved in the rotarod test at the end of each week during a 3 week ES treatment period (*n* = 3). The maximum F) time and G) distance achieved in the treadmill test at the end of each week during a 3 week ES treatment period (*n* = 3). The week 1 to week 3 change in the H) rotarod test time, I) rotarod test RPM, J) treadmill test time and K) treadmill test distance for the three groups (*n* = 3). Behavioral tests were conducted on three biologically distinct mice of the same age, and the average of two tests per mouse was recorded. The data are expressed as the mean ± s.d. *p < 0.05, **p < 0.005, ***p < 0.0005.

Next, we evaluated the effect of ES on ameliorating CMT symptoms in heterozygous Tr‐J mice. While homozygous Tr‐J mice exhibit severe demyelination and rarely survive to adulthood, heterozygous mice have been regarded as demonstrating more typical disease symptoms with human demyelinating CMT,^[^ ^36^ ^]^ exhibiting gradual demyelination and deterioration in physical performance.^[^ ^15b,c,^, ^37^ ^]^ Cuff electrodes were implanted onto the sciatic nerves of Tr‐J mice (**Figure** **5**A), and ES was provided at the beginning of each week for 3 consecutive weeks (Figure 5B). At the end of each week, rotarod and treadmill tests were performed as representative behavioral evaluations for physical balance and running endurance, respectively (Figure 5C). Additional details regarding the cuff electrode and its implantation are described in the Experimental Section, and shown in Figure S5, Supporting Information.

Compared with age‐matched WT mice, the control Tr‐J mice had significantly impaired physical balance, as assessed by the maximal time and rotations per minute (RPM) achieved on the rotarod before falling (Movie S1, Supporting Information). After ES treatment, the Tr‐J mice demonstrated progressive week‐by‐week improvements in both of these physical balance parameters. At the end of the last week of treatment, the ES‐treated Tr‐J mice were able to remain on the rotarod for 54 s (46%) longer and achieve 5.4 higher RPM (24%) than control Tr‐J mice (Figure 5D,E). Similarly, the Tr‐J mice had significantly impaired running endurance, as assessed by the maximal time on the treadmill and the distance covered (Movie S2, Supporting Information). These parameters also showed progressive improvement after ES treatment during the same period. At the end of the last week of treatment, the ES‐treated Tr‐J mice were able to remain on the treadmill for 85 s (16%) longer and covered 37 m (29%) more distance than Tr‐J control mice (Figure 5F,G).

Another critical observation regarding behavioral phenotype was the extent of improvement between the 1st and 3rd weeks of the rotarod and treadmill tests. Compared with the improvements exhibited by the WT mice in physical balance (Figure 5H,I) and running endurance (Figure 5J,K) within this period—presumably due to habituation—the Tr‐J control mice exhibited significantly lower degrees of improvements within the same period, suggesting an impediment in increasing their physical performance ceiling without any intervention. Notably, compared with the Tr‐J control mice, the ES‐treated Tr‐J mice showed significantly higher improvements in physical balance (Figure 5H,I) and running endurance (Figure 5J,K). It should be noted that the ES‐treated Tr‐J mice showed the highest improvements between the 1st and 3rd weeks across all three groups. These results support the potential of ES in promoting rehabilitation of behavioral phenotypes of Tr‐J mice, hopefully translating toward a clinical CMT intervention.

### 2.7 Implantable ES Improves Myelination in the Sciatic Nerve of Tr‐J Mice

**Figure 6:**
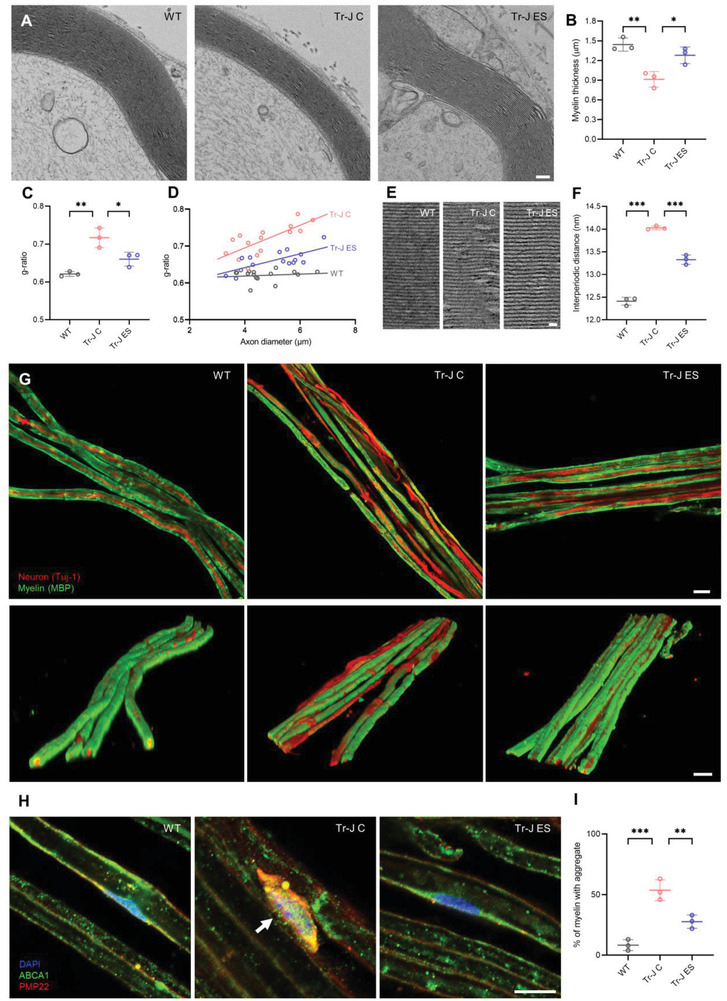
ES ameliorates demyelination and PMP22/ABCA1 distribution abnormality in vivo. A) Representative transmission electron microscopy (TEM) images of cross‐sections of sciatic nerves. Scale bar, 500 nm. B) Myelin thickness measured from TEM images (*n* = 3). C) The g‐ratio measured from TEM images (*n* = 3). D) Relationship between g‐ratio and axon diameter (*n* = 3). E) Representative TEM images at higher magnification showing the interperiodic distance. Scale bar, 20 nm. F) Measurement of the interperiodic distance from TEM images (*n* = 3). G) Representative 2D and 3D confocal images of teased longitudinal sections of sciatic nerves immunostained against MBP and Tuj‐1. Scale bar, 10 µm. H) Representative images of longitudinal sections of sciatic nerves immunostained against PMP22 and ABCA1. The white arrow points to PMP22/ABCA1 aggregation in the perinuclear region. I) Percentage of myelin segments with PMP22/ABCA1 aggregates in the perinuclear region (*n* = 3). For myelin thickness, g‐ratio, and interperiodic distance, the average of five measurements was taken from the TEM images of three biologically distinct samples. The percentage of myelin segments with aggregates was counted from 20× images of three biologically distinct samples per condition. The data are expressed as the mean ± s.d. *p < 0.05, **p < 0.005, ***p < 0.0005.

To evaluate the effect of ES treatment on in vivo myelination at the end of the 3 weeks period, sciatic nerves were isolated from the mice, and their myelination was evaluated via transmission electron microscopy (TEM) imaging of cross‐sections and 3D confocal imaging of longitudinal sections of the sciatic nerves. Using the TEM images, we compared the myelin thickness, myelin layer compactness, and the g‐ratio, which is the ratio of the inner diameter (axon only) to the outer diameter (axon and myelin combined) of each neuron. The g‐ratio is a useful metric to assess the axonal myelination condition as an indicator of CMT1 disease, as it increases significantly during demyelination, indicating thinner myelin relative to the thickness of the axon.^[^ ^38^ ^]^ The TEM images of the sciatic nerves (**Figure** **6**A) showed that the Tr‐J mice had 36.7% lower myelin thickness (Figure 6B) and a 15.5% higher g‐ratio (Figure 6C) than the WT mice. After ES treatment, surprisingly, the Tr‐J mice showed a 40.1% improvement in myelin thickness (Figure 6B), and the g‐ratio was restored by 7.9% (Figure 6C). It should be noted that while the WT sciatic nerves presented with consistent g‐ratios for different axon diameters, the Tr‐J sciatic nerves showed increased g‐ratios that depended on the axon diameter, indicating more demyelination in thicker axons. However, ES stimulation alleviated the change in the g‐ratio versus axon diameter, suggesting that the intervention reduced the extent of demyelination in all axon diameters of Tr‐J sciatic nerves (Figure 6D). Furthermore, the interperiodic distance of the myelin in the Tr‐J mice was significantly reduced by ES, implying that ES not only improved myelin thickness but also made myelin layers more compact (Figure 6E,F). To obtain longitudinal sections of images, individual fibers in the sciatic nerves were teased out and immunostained. Longitudinal sections of 2D and 3D confocal images revealed that the Tr‐J mice had a greater prevalence of demyelinated or thinly myelinated axons than the WT mice. In contrast, upon ES intervention, the Tr‐J mice showed marked improvement in axon myelination, closer to the myelin phenotype of the WT mice (Figure 6G and Movie S3, Supporting Information).

Finally, we evaluated the colocalization of PMP22 and ABCA1 in the myelin segments of the sciatic nerves via confocal imaging (Figure 6H and Figure S6, Supporting Information). While perinuclear PMP22/ABCA1 aggregates were mostly absent in WT myelin segments, the Tr‐J sciatic nerves contained numerous myelin segments with such aggregates (marked by white arrow in Figure 6H) (8.3% in WT vs 53.7% in Tr‐J control). In the myelin of Tr‐J ES mice, this localization abnormality was significantly ameliorated, as the percentage of myelin segments containing perinuclear PMP22/ABCA1 aggregates was decreased (53.7% in Tr‐J control vs 27.7% in Tr‐J ES) (Figure 6I). These results confirmed that ES ameliorates demyelination as well as perinuclear PMP22/ABCA1 aggregation in the peripheral neurons of Tr‐J mice.

## 3 Discussion

In this study, we introduced an electroceutical treatment for demyelinating CMT disease. Although there have been numerous efforts to treat CMT disease, including ascorbic acid, high‐fat diet, and phosphatidylcholine supplementation,^[^ ^4^, ^5^ ^]^ unfortunately, an approved treatment for CMT disease is yet to be found. While the previous biochemical interventions did not result in an approved treatment, a biophysical intervention for CMT disease had not been reported. Electroceuticals are biophysical interventions that can mimic the nervous system communication, making them an appealing neuromodulation strategy.^[^ ^39^ ^]^ ES has been routinely applied toward diverse CNS neuropathies, such as Parkinson's disease, epilepsy, and spinal cord injury.^[^ ^9^ ^]^ As a notable study, ES showed beneficial effects to improve the symptoms in patients of demyelinating neuropathies of the CNS as early as 1977.^[^ ^40^ ^]^ In aspect of the PNS, although electroceuticals have also shown promising results, particularly in neuroregeneration,^[^ ^13^ ^]^ its efficacy toward peripheral demyelinating neuropathies had remained unexplored. Recently, we have shown that an electroceutical approach enhances myelination in the WT mouse DRG.^[^ ^14^ ^]^ Here, we have advanced the electroceutical application to a peripheral demyelinating neuropathy using a representative animal model, evaluating its effectiveness based on improvements in PNS myelination and physical performance, while also providing mechanistic insight into the biomolecular changes that led to these improvements.

Using a neuron‐SC coculture, we carried out in vitro screening, which showed that ES at 20 Hz for 1 h was optimum for improving myelination. Further validation of this ES condition was carried out using biomimetic ex vivo DRG explants. One interesting observation was the greater extent of myelination improvement in the distal region of the DRG relative to the proximal. This might be due to the AP‐mimicking ability of ES.^[^ ^41^ ^]^ The AP along an axon can be detected by nearby SCs, which subsequently initiates a pro‐myelinating response.^[^ ^42^ ^]^ Since the AP becomes weaker as it propagates along the axon, the pro‐myelinating effect can deteriorate in the distal region of the neuron. The ES might have mimicked the effect of the AP on the SCs, which consolidated the pro‐myelinating effect in the distal region of the neuron. We also analyzed the sciatic nerves via TEM and 3D confocal imaging, and found that a 3 week electroceutical treatment restored the myelin thickness, compaction, and membrane integrity closer to WT levels, thus indicating improved in vivo myelination.

Another highlight of this study is the mechanistic insight toward understanding the biomolecular changes that led to the improvement in myelination. ES reduced the retention of myelin protein PMP22 around the perinuclear region of Tr‐J myelin. This effect might have been driven by calcium ion transients upon ES, since proper calcium signaling can enhance the posttranslational modification of PMP22 by the calnexin‐calreticulin chaperones in the ER.^[^ ^27^, ^43^ ^]^ This is also supported by our finding that ES attenuated the ER stress in Tr‐J, likely due to the alleviation of the intrinsic conformational instability and mistrafficking of Tr‐J PMP22.^[^ ^44^ ^]^ The improvement of PMP22 distribution was accompanied by improvements in the de novo biosynthesis and distribution of cholesterol in the Tr‐J myelin membrane. This effect might play an important role in clinical translation, since it potentially eliminates the need for exogenous lipid supplementation, thus avoiding the probable systemic side effects of recent approaches using lipid supplements.^[^ ^5^ ^]^ The step‐by‐step mechanism via which ES led to these biomolecular changes could be elucidated in future work.

Along with improving myelination, ES resulted in gradual rehabilitation of the behavioral phenotype of the mice across the 3 week electroceutical treatment period, demonstrating the extent of the therapeutic efficacy of this approach. The thicker myelin sheaths around the peripheral neurons could have enabled better signal propagation to the skeletal muscle of the mice, enabling better physical performance.^[^ ^45^ ^]^ In addition to peripheral demyelination and behavioral abnormalities, Tr‐J mice exhibit other neuromuscular defects, such as skeletal muscle atrophy and fragmentation of the post‐synaptic terminal at the neuromuscular junction. However, Tr‐J mice start to exhibit these neuromuscular defects when they are significantly older, compared to their age when they start to exhibit peripheral demyelination and behavioral abnormalities (10 months vs 2–6 months).^[^ ^15c^ ^]^ The age of the mice in this study was chosen such that they are old enough to exhibit peripheral demyelination, but young enough to maintain SC plasticity.^[^ ^46^ ^]^ Therefore, the improvements of the aforementioned neuromuscular defects after ES were not investigated in this study. Given that ES improved peripheral myelination and behavioral phenotypes in 6 months old Tr‐J mice, further study to investigate the effects of ES on neuromuscular defects in older Tr‐J mice (≥10 months) would be justifiable.

Although electroceutical applications have predominantly focused on CNS neuropathies thus far, their prospect toward PNS neuropathies is potentially more appealing. One limitation of applying ES in the CNS is the unwanted spread of the impulses via the branched neuronal connections. In contrast to the CNS, neuronal connections in the PNS are straightforward and unidirectional, making it more feasible to target end organs or nerve bundles via electroceuticals.^[^ ^47^ ^]^ The practical aspects of electroceutical application in the PNS have also progressed significantly. For example, the development of miniature, wireless, and flexible neuromodulation devices has made the surgical implantation less invasive.^[^ ^48^ ^]^ This is aided by recent advances in the development of biocompatible, soft, and conductive polymers, which reduce mechanical mismatch with the nerves and thereby facilitate biointegration.^[^ ^49^ ^]^

## 4 Conclusion

In conclusion, the present study demonstrates that an electroceutical approach ameliorates key phenotypes in a mouse model of CMT disease, a peripheral demyelinating neuropathy. Using an in vitro screening step, ES at 20 Hz for 1 h is identified as the optimal condition to improve myelination. The ES‐mediated myelination improvement is further verified at the ex vivo stage. Biomolecular analysis reveals that ES restores the myelin membrane integrity by ameliorating characteristic abnormalities of CMT disease, such as PMP22 and cholesterol distribution in the myelin membrane. ES is then provided in vivo across a 3 week period via implantable electrodes onto the sciatic nerves, which results in a gradual rehabilitation in the behavioral phenotype of the mice. After ES, the analysis of in vivo myelination shows that ES improves myelin thickness, compactness, and membrane integrity. This study is expected to lay the groundwork for the development of electroceutical treatments for peripheral demyelinating neuropathies, which can be realized via further elucidation of the mechanism and the maturation of neuromodulation technologies.

## 5 Experimental Section

### Animal Usage and Ethics Approval

Wild‐type (WT; C57BL/6J) and heterozygous Tr‐J (B6.D2‐Pmp22^Tr‐J^/J) mice were purchased from Jackson Laboratory. All animal experiments and procedures were approved by the Institutional Animal Care and Use Committee (IACUC) affiliated with the Laboratory Animal Resource Center (LARC) of DGIST (DGIST‐IACUC‐20062502‐01).

### Preparation of Matrigel‐Coated Coverslips

13 mm plastic coverslips (Thermo Scientific; product number 174950) were placed in individual wells of a 24‐well plate (Corning; product 3526). 50% Matrigel (Corning; product 356231) was prepared by diluting with prefiltered cold Dulbecco's modified Eagle medium (DMEM). Each coverslip was coated in a thin layer of 50% Matrigel and allowed to polymerize by incubating at 37 °C for 1 h.

### Neuron‐SC Coculture

Neuron‐SC coculture was carried out as described previously.^[^ ^24b^ ^]^ Briefly, DRGs were isolated from the spinal columns of WT and Tr‐J mouse embryos at E13.5 (Figure S1A,B, Supporting Information). Ten isolated DRGs were transferred into a 15 mL conical tube containing 5 mL collagenase diluted in Dulbecco's phosphate buffered saline (DPBS, final concentration 0.5 mg mL^−1^), followed by incubation at 37 °C for 90 min. 2.5 mL DPBS and 25 µL of 2.5% Trypsin/ethylenediaminetetraacetic acid was then added, followed by incubation at 37 °C for 15 min. 2.5 mL DMEM containing 10% fetal bovine serum was then added to the mixture, followed by a final incubation period of 5 min at 37 °C. Finally, DRG‐dissociated cells were separated by centrifugation at 1300 RPM for 3 min.

For obtaining SCs, sciatic nerves were isolated from 4 days old WT and Tr‐J mouse pups (Figure S1D, Supporting Information). The dissociation steps were carried out as described above, followed by separation by centrifugation at 3000 RPM for 1 min.

The purification step to obtain primary neurons from DRG‐dissociated cells was carried out as described previously.^[^ ^50^ ^]^ Briefly, DRG‐dissociated cells suspended in growth medium were added at a concentration of 50 000 cells per well onto Matrigel‐coated coverslips. Purification step was started the following day by adding an inhibitor cocktail of Ara‐C (final concentration 0.5 × 10^−6^ m), Floxuridine and Uridine (final concentration 20 × 10^−6^ m for each). This inhibitor treatment was carried out for 72 h to obtain purified neurons (Figure S1C, Supporting Information). Following a week of culture in growth medium, SCs (Figure S1E, Supporting Information) isolated from mouse pup sciatic nerves were added at 7 DIV with a concentration of 50 000 cells per well, suspended in differentiation medium. This coculture was continued for a week before changing the medium to myelination medium. ES was provided after 1 day of acclimatization in the myelination medium (15 DIV). Following ES, the myelination was allowed to continue before further analyses (Figure S1F, Supporting Information). The compositions of the growth, differentiation and myelination media are provided in Table S1, Supporting Information.

### Whole DRG Explant Culture

Whole DRGs were extracted as described previously.^[^ ^51^ ^]^ Briefly, the spinal columns were isolated from 8 weeks old WT or Tr‐J mice. The DRGs from the L1‐L6 were extricated and residual meninges were removed. During the procedure, the DRGs were suspended in Hank's balanced salt solution (Welgene; catalog number LB 003–04) before seeding on the Matrigel‐coated coverslip. After seeding, the DRGs were allowed to attach by incubating the 24‐well plate at 37 °C for 2 h. This was followed by addition of growth medium, carefully pipetted along the walls of the plate. The DRGs were incubated in growth medium for a week. The medium was then changed to differentiation medium in which the DRGs were incubated for a further 1 week. Finally, the medium was changed to myelination medium. After allowing the DRGs to acclimatize to the new medium for a day, ES session was carried out and myelination was allowed to continue before further analyses (Figure S4A, Supporting Information). The compositions of the growth, differentiation, and myelination media are provided in Table S1, Supporting Information.

### In Vitro and Ex Vivo Stimulation

The ES device was designed to match the dimensions of a 100 mm petri dish, and fabricated out of polyetheretherketone (PEEK) via computerized numerical control (CNC) machining. PEEK had excellent resistance toward oxidation and heat, making it suitable to fabricate ES devices compatible with biological applications as well as autoclaving. The ES device was consisted of three parts: the lower chamber, the gold‐plated electrodes, and the lid (Figure S2A, Supporting Information). The lower chamber has six shallow wells to securely hold coverslips. During stimulation, the coverslips containing the neuron‐SC cocultures or DRGs were positioned within the wells of the ES device, and 15 mL myelination medium was added to the device. A uniform 50 mV mm^−1^ electric field was generated across each coverslip (Figure S2B, Supporting Information) and maintained for 1 h. For the in vitro neuron‐SC coculture, biphasic signals of 5, 20, 100, and 500 Hz were applied; for the ex vivo DRG explant, a biphasic signal of 20 Hz was applied. The electrode configuration was selected based on the balance of the electric field uniformity and the high‐throughput capability (Figure S2C, Supporting Information). The impedance (Figure S2D, Supporting Information) and the long‐term reliability (Figure S2E, Supporting Information) of the electrodes were also within satisfactory limits. The temperature change within the medium was also negligible, even at relatively strong electric fields (Figure S2F, Supporting Information). After the ES session, the coverslips were taken out of the ES device and placed back into the 24‐well plates.

### In Vivo Stimulation

The schedule followed for electrode implantation, recovery, ES treatment, and behavioral tests is provided in Figure S5A, Supporting Information. Nerve cuff electrodes were purchased from Microprobes. The electrodes had an inner diameter of 0.5 mm, with three platinum contacts of 100 µm thickness spaced 3 mm apart (Figure S5B,C, Supporting Information). Electrodes were implanted onto the sciatic nerves of 6 months old Tr‐J mice (Figure S5D, Supporting Information) and the lead wires were routed via the subcutaneous layer to bring the head port out via the scalp. The head port was secured to the head using dental putty (Figure S5E, Supporting Information). Before ES, the mice were given 1 week to recover from the implantation surgery and a further 1 week for rotarod and treadmill training. Proper attachment of the electrode to the sciatic nerve was confirmed via twitching of the leg upon short pulses of stimulation. ES was provided once per week—each stimulation lasting 30 min—on the first day of each week for 3 consecutive weeks. During stimulation, the mouse was under isoflurane‐induced anesthesia (nasal administration). The WT and Tr‐J C mice were also under isoflurane‐induced anesthesia for the same duration. The stimulator connected to the electrode was Model 4100 from A‐M Systems and the signal was generated at 20 Hz, 0.5 V and a pulse width of 100 ms. Behavioral tests were carried out every week and myelination assessment were done at the end of the 3 weeks period.

### Behavior Test

Mice were trained on the rotarod and treadmill three times in a week (on alternate days) before carrying out tests. The tests were then carried out on the last day of the same week during which ES was provided, for 3 consecutive weeks. For the rotarod test, a constant acceleration in RPM was used.^[^ ^52^ ^]^ Initial spin was set to 10 RPM and programmed to increase to 40 RPM gradually over a 5 min period. RPM and time were recorded at the point where the mouse fell off from the rotarod. For the treadmill test, initial speed was set to 20 cm s^−1^, which was sustained for 5 min. Then, the speed was increased by 5 cm s^−1^ every minute till total speed reached 50 cm s^−1^. The test was concluded if the mouse reached the exhaustion zone five times in under a minute. Each test was conducted twice for each mouse (with a rest period of 15 min) and the average value was recorded

### Immunostaining and Imaging

Neuron‐SC coculture and whole DRG explants were immunostained at 35 DIV. Fixation was carried out via 4% paraformaldehyde (PFA) treatment for 15 min at room temperature (RT). This was followed by treatment with blocking buffer (1X PBS, 0.3% Triton X‐100, and 5% Normal Donkey Serum) for 1 h at RT. Primary antibodies diluted in antibody dilution buffer (ADB; 1X PBS, 0.3% Triton X‐100, and 1% Normal Donkey Serum) were then treated overnight at 4 °C. Then, secondary antibodies diluted in ADB were treated for 2 h at RT, protected from light. Lastly, where needed, Hoechst (1X) was treated for 15 min at RT.

Immunostaining of sciatic nerves was carried out by first surgically removing the sciatic nerves from WT or Tr‐J mice. These were fixed using 4% PFA treatment for 2 h at RT. Individual axons were then teased from these nerves using a pair of sharp surgical tweezers and a needle. Teased axons were placed on top of Superfrost slides (Thermo Fisher Scientific; product code: 10149870) and allowed to attach sufficiently. Permeabilization was carried out by acetone treatment for 10 min at −20 °C. Primary antibodies diluted in ADB were treated overnight at 4 °C. Secondary antibody and Hoechst treatments were done as mentioned above.

Antibodies: rabbit monoclonal anti‐MBP (Abcam; catalog number ab40390; used at 1:200 dilution), mouse monoclonal anti‐Tuj1 (Abcam; catalog number ab78078; used at a 1:1000 dilution), rabbit polyclonal anti‐ABCA1 (Invitrogen; catalog number PA1‐32129; used at 1:100 dilution), rabbit polyclonal anti‐Calnexin (Invitrogen; catalog number PA5‐34754; used at 1:200 dilution), mouse monoclonal anti‐PMP22 (Santa Cruz Biotechnology; catalog number sc‐515199; used at 1:50 dilution), donkey anti‐rabbit Alexa fluor 488 (Jackson ImmunoResearch; catalog number 711‐545‐152; used at 1:200 dilution), donkey anti‐mouse Alexa fluor 594 (Jackson ImmunoResearch; catalog number 715‐585‐151; used at 1:200 dilution), donkey anti‐rabbit Alexa fluor 594 (Jackson ImmunoResearch; catalog number 711‐585‐152; used at 1:200 dilution) and donkey anti‐mouse Alexa fluor 647 (Jackson ImmunoResearch; catalog number 715‐605‐150; used at 1:200 dilution).

Imaging was performed with Nikon A1R confocal microscope, via the NIS‐Elements software available from Nikon.

### ToF‐SIMS Imaging

Chemical fixation was carried out by treatment with 10% formalin solution (Sigma Aldrich) for 10 min followed by 2% glutaraldehyde (Electron Microscopy Sciences) for 15 min, both at RT. Then, post‐fixation was carried out using 0.4% osmium tetroxide treatment for 15 min at RT. Prior to imaging, the cells were treated with air plasma (CUTE, Femto Science Inc., Republic of Korea) at 1.1–1.3 Torr, 50 kHz, 100 W, and 70 sccm for 5 min.

ToF‐SIMS analysis was done via ToF‐SIMS model 5–100 from ION‐TOF, Germany, using a pulsed 30 keV Bi_3_^+^ primary ion beam in delayed extraction mode for positive ions. ToF‐SIMS images were obtained from fixed cells in an area of 300 × 300 µm^2^ with 256 × 256 pixels. Internal calibration was performed using the peaks of CH_3_^+^, Na^+^, C_2_H_3_^+^, C_3_H_5_^+^, and C_4_H_7_^+^. For charge compensation, low‐energy electrons were provided to the surface using an electron flood gun.

ImageJ (IJBlob library) was used for fragment size quantification.^[^ ^53^ ^]^ ToF‐SIMS images were converted to gray images and then thresholding was applied to subtract the background with low intensity. This was followed by particle size analysis to measure the area of the fragments. For each condition, images from three biologically distinct samples were used.

### RT‐qPCR

The mRNA extraction was carried out using RNeasy Mini Kit (Qiagen; catalog number: 74106). This was followed by cDNA synthesis as soon as possible, using AccuPower RT Premix (Bioneer; catalog number: K‐2041‐B). The temperature cycling was done using Biometra Tone (Analytik Jena), with temperature and duration as recommended for the AccuPower RT Premix. RT‐qPCR was carried out on three biologically distinct samples from each group. Three technical replicates were prepared for each sample and average *C*_T_ value was taken for further comparison. Applied Biosystems StepOnePlus was used for thermal cycling and *C*_T_ measurements, and the software StepOne v2.3 available from Applied Biosystems was used to directly calculate the fold changes in the Trembler‐J control and ES, compared to the wild‐type control. The sequences of the primers used are provided in Table S2, Supporting Information.

### TEM Imaging of Sciatic Nerves

Fixation was carried out on freshly dissected sciatic nerves using a 2.5% glutaraldehyde treatment at 4 °C for 2 h, followed by 1% osmium tetroxide treatment at 4 °C for 2 h. Next, serial dehydration was carried out using 30% (4 °C), 50% (4 °C), 70% (4 °C), 80% (4 °C), 90% (RT), 95% (RT), and 100% (RT) ethanol, each treatment lasting 15 min. This was followed by rinsing in 100% propylene oxide three times at RT, each rinse lasting 20 min. EMbed 812 Kit (Electron Microscopy Sciences; catalog number: 14120) was used for the subsequent embedding stages. The sciatic nerve was left in a 1:1 mixture of propylene oxide and resin for 3 h. The sciatic nerve was taken out of this mixture and put inside soft Epon mixture overnight, followed by incubation for 3 h in medium Epon mixture. The sciatic nerve was then put inside embedding mold containing hard Epon mixture and then polymerized at 60 °C overnight. 70 nm sections were obtained using an ultramicrotome (Leica; model number: EM UC7) and post staining was carried out using lead citrate and uranyl acetate treatment. Imaging was then carried out via FEI Tecnai G2 F20 TWIN TMP. The g‐ratio was calculated from the TEM images as the ratio of the inner diameter (only the axon) to the outer diameter (axon and myelin combined) of each neuron. Measurements were taken from three images for each group.

### Statistical Analysis

Values were expressed as mean ± s.d. Statistical significance was calculated with GraphPad Prism software. One‐way analysis of variance (ANOVA) followed by Tukey's comparison test was used to compare the groups. For Figure 4, two‐way ANOVA followed by Tukey's comparison test was used to compare the groups. The *p*‐values less than 0.05 were considered significant differences.

## Conflict of Interest

The authors declare no conflict of interest.

## Supporting information

Supporting information

Supplemental Movie 1

Supplemental Movie 2

Supplemental Movie 3

## Data Availability

The data that support the findings of this study are available in the supplementary material of this article.
