# Supplementary material for: Implantable Electroceutical Approach Improves Myelination by Restoring Membrane Integrity in a Mouse Model of Peripheral Demyelinating Neuropathy
Source: Adv Sci (Weinh). 2022 Aug 17;9(32):2201358. doi: 10.1002/advs.202201358 (PMC9661852; doi:10.1002/advs.202201358)
Supplement: Supplementary file 1 — Supporting information [file ADVS-9-2201358-s003.pdf]

ADVANCED SCIENCE
Open Access

Supporting Information

for *Adv. Sci.*, DOI 10.1002/advs.202201358

Implantable Electroceutical Approach Improves Myelination by Restoring Membrane Integrity in a Mouse Model of Peripheral Demyelinating Neuropathy

*Aseer Intisar, Hyun Young Shin, Woon-Hae Kim, Hyun Gyu Kang, Min Young Kim, Yu Seon Kim, Youngjun Cho, Yun Jeoung Mo, Heejin Lim, Sanghoon Lee, Q. Richard Lu, Yun-Il Lee and Minseok S. Kim**

Supporting Information

Implantable Electroceutical Approach Improves Myelination by Restoring Membrane Integrity in a Mouse Model of Peripheral Demyelinating Neuropathy

*Aseer Intisar, Hyun Young Shin, Woon-Hae Kim, Hyun Gyu Kang, Min Young Kim, Yu Seon Kim, Youngjun Cho, Yun Jeoung Mo, Heejin Lim, Sanghoon Lee, Q. Richard Lu, Yun-Il Lee, and Minseok S. Kim**

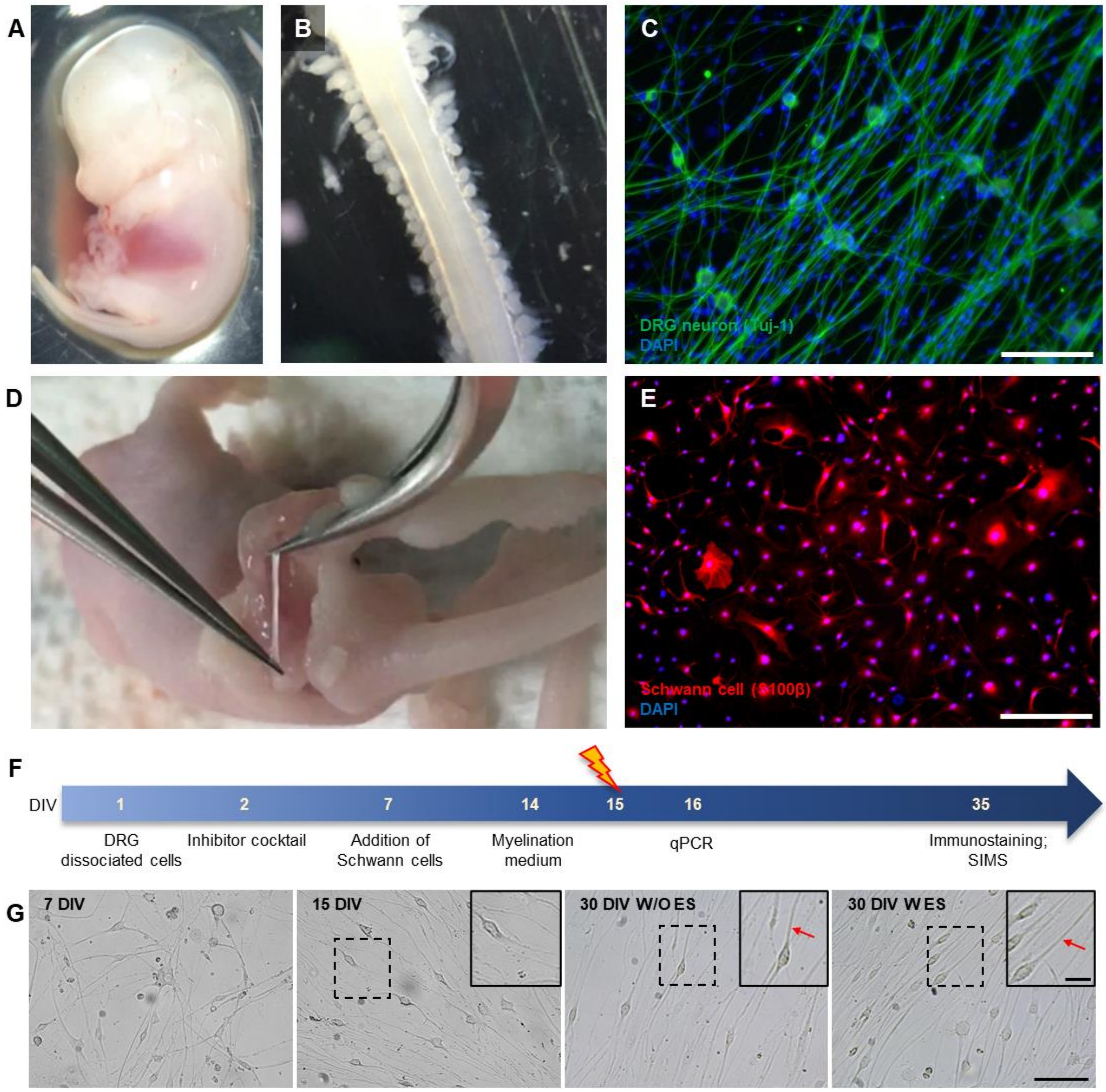

Figure S1. Isolation of cells and the timeline of *in vitro* coculture. (**A**) Mouse embyro at E13.5. The spine is extracted carefully to retain as many intact dorsal root ganglia (DRGs) as possible. (**B**) An extracted spine showing the spherical DRGs on either side. These DRGs are further dissociated, and the dissociated cells are seeded onto a Matrigel-coated surface. Neurons are purified using a 72-h cocktail treatment. (**C**) Confocal image of immunostained DRG-dissociated cells after the purification step. A high density of neuronal cells remains after the purification step. Scale bar, 100 µm. (**D**) Extraction of sciatic nerves from 4-week-old mouse pups. These sciatic nerves are then dissociated, and the SCs are isolated. (**E**) A confocal image of immunostained Schwann cells (SCs) (isolated from mouse pup sciatic nerves) at 1 DIV. Scale bar, 100 µm. (**F**) Timeline of neuron-SC coculture. (**G**) Representative bright-field images of the purified neurons and the Trembler-J neuron-SC coculture. From left to right: purified neurons at 7 DIV (just before the addition of SCs),

coculture at 15 DIV (just before the start of ES), and coculture at 30 DIV without and with ES. The image in the inset shows the enlarged version of the region marked by the dotted rectangle. In the cocultures at 30 DIV, myelin is likely to be more visible as dark streaks (shown by the red arrows in the insets) compared to that at 15 DIV. Scale bars, 100 μm.

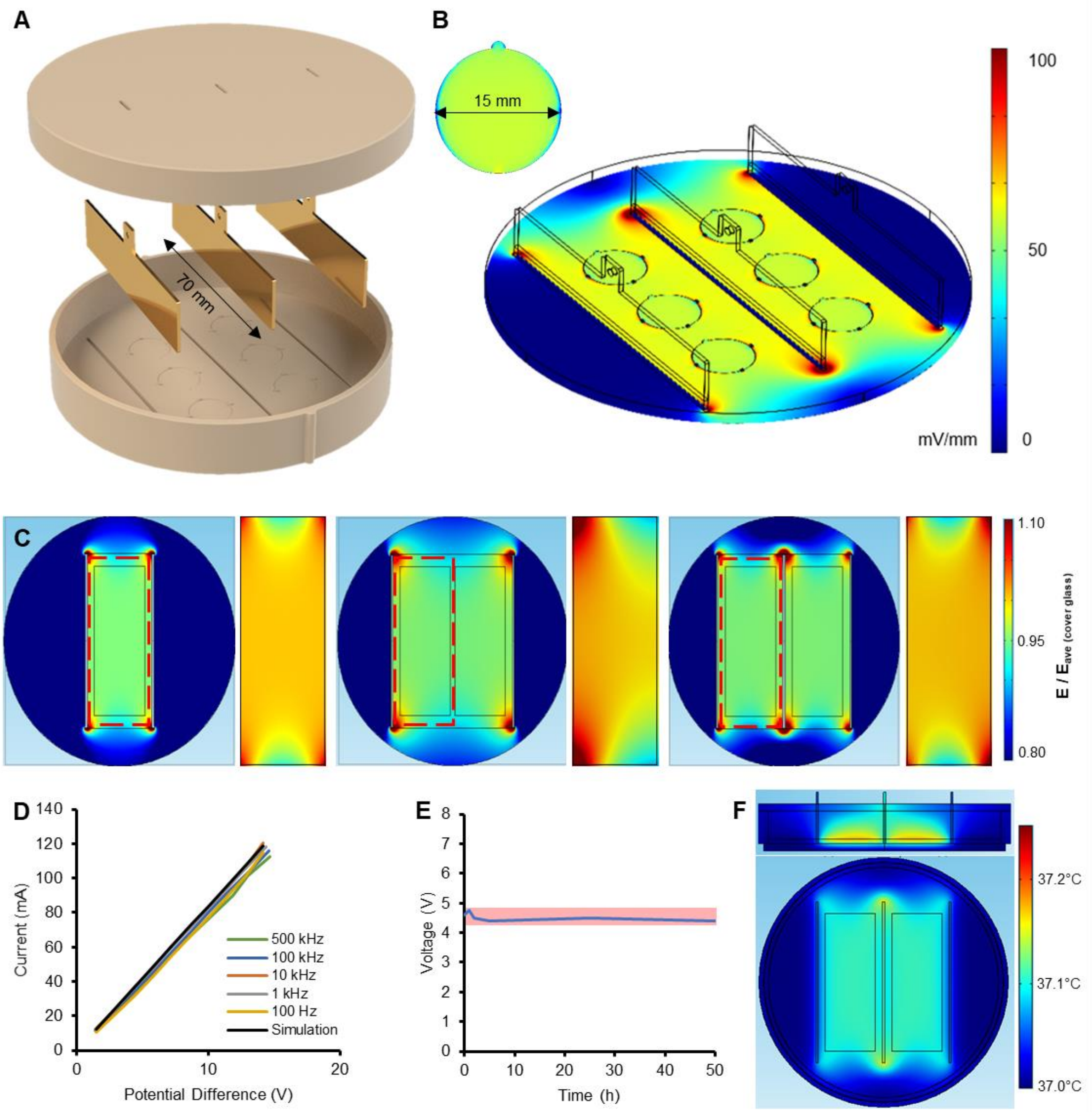

Figure S2. Electrical stimulation platform for *in vitro* and *ex vivo* studies. (**A**) The overall configuration of the platform, showing the lower chamber, which holds six 15 mm coverslips, three gold-plated electrodes and the lid with slits to hold the electrodes equidistant and parallel. (**B**) COMSOL simulation showing the uniformity of the electric field while generating 50 mV/mm within the wells that hold the coverslips. The inset shows a closer look at the uniformity within a single well. (**C**) COMSOL simulation showing the uniformity of the distribution of the electric field generated when using (from left to right): two electrodes carrying opposite charges spaced one column apart (99.1% uniformity in ROI), two electrodes carrying opposite charges spaced two columns apart (97.7% uniformity in ROI) and three electrodes spaced one column apart, with the two electrodes on either side carrying the same charge and the one in the middle carrying the opposite charge (98.8% uniformity in ROI). (**D**)

Impedance measurement at different frequencies shows that it is comparable to the simulated value across all frequencies. (**E**) Reliability test performed by maintaining 4.5 V for 50 h continuously (phosphate buffered saline used as the medium). The voltages measured at different time points are all within a narrow range (± 0.13 V). (**F**) Temperature change while maintaining a 500 mV/mm electric field, which is 10 times stronger than the electric field used during *in vitro* and *ex vivo* stimulation (phosphate buffered saline used as medium). The temperature was not significantly changed.

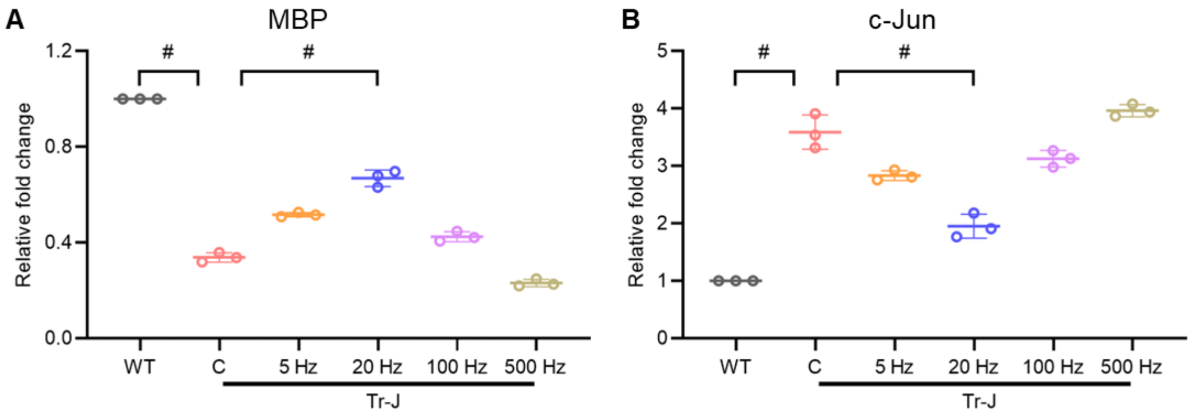

Figure S3. Changes in the expression of genes related to myelination. Quantitative reverse transcription polymerase chain reaction (RT-qPCR) analysis for the expression of (**A**) myelin basic protein (MBP) (n = 3) and (**B**) c-Jun (n = 3) in the wild-type (WT) and Trembler-J (Tr-J) neuron-Schwann cell cocultures, with the latter treated with electrical stimulation at 5, 20, 100 and 500 Hz. The data are expressed as the mean ± s.d. # $p < 0.0001$.

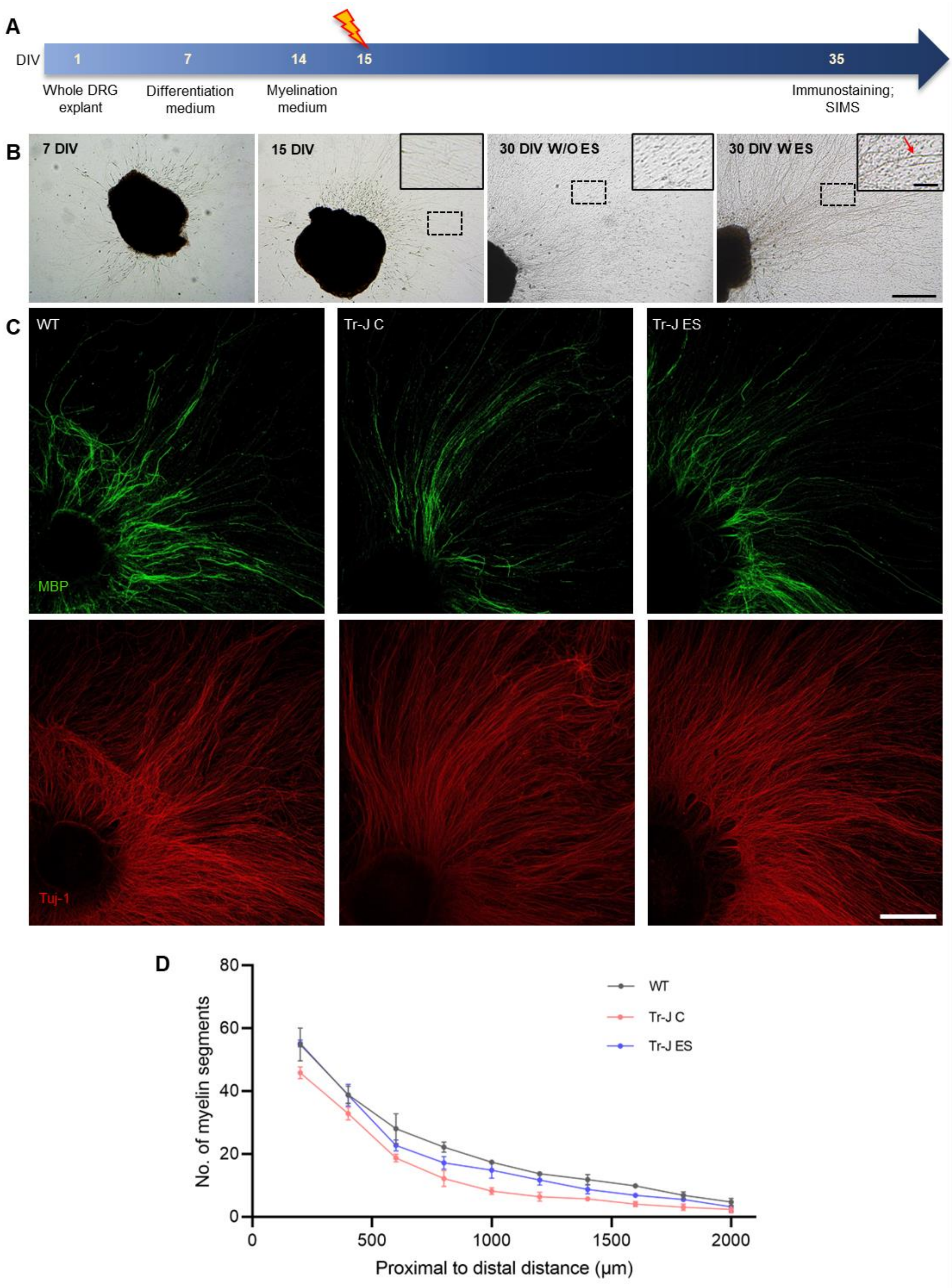

Figure S4. Timeline of *ex vivo* experiments and representative images of DRG myelination. (**A**) Timeline of *ex vivo* DRG explant culture. Upon DRG seeding, a one-week period was allotted for growth, followed by a one-week period for differentiation. This was followed by the addition of myelination medium at 14 DIV. Electrical stimulation (ES) was

provided at 15 DIV, and the DRG explant was cultured in myelination medium until further analysis. (**B**) Representative bright-field images of the Trembler-J DRG explant. From left to right: DRG at 7 DIV (just before the addition of differentiation medium), DRG at 15 DIV (just before the start of ES), and DRG at 30 DIV without and with ES. The image in the inset shows the enlarged version of the region marked by the dotted rectangle. In the ES DRG at 30 DIV, myelin is visible as dark streaks (one such segment is shown by the red arrow in the inset). These dark streaks are not clearly visible in the DRG at 15 DIV. Scale bars, 500 μm (main) and 100 μm (inset). (**C**) Individual MBP and Tuj-1 channels of representative images of DRG explants at 35 DIV. Scale bar, 500 μm. (**D**) Numbers of myelin segments in the DRG explant in each 200 μm radial region, starting from the edge of the explant. It was measured using the MBP (green) channel from 4× images of three biologically distinct samples per condition. The data are expressed as the mean ± s.d.

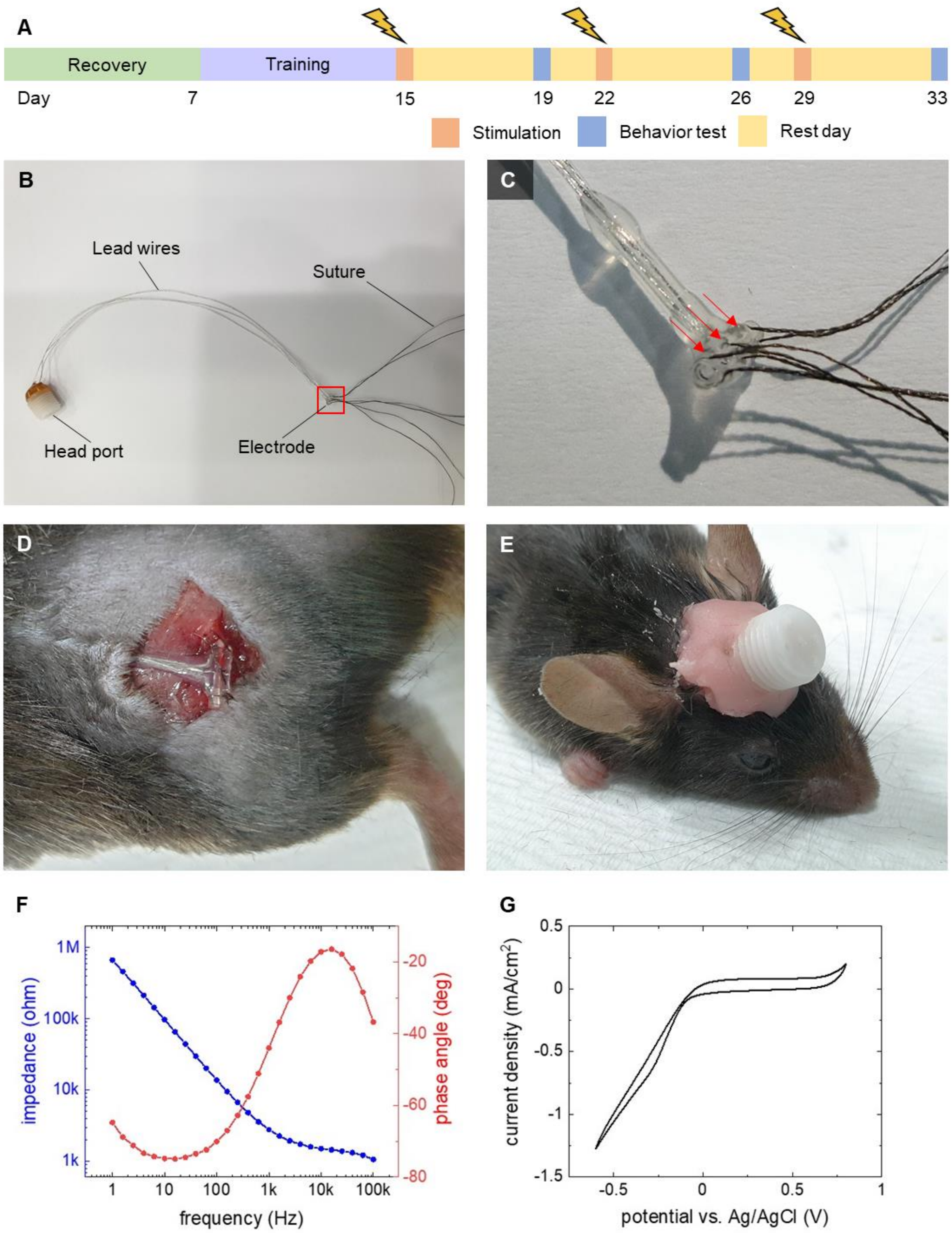

Figure S5. ***In vivo* electrical stimulation (ES) and electrode characterization.** (**A**) Timeline of ES and the behavioral tests. One week after the surgery, all mice were trained on the rotarod and treadmill for one week. Following the training period, mice were administered ES at the beginning of every week for three consecutive weeks, and behavioral tests were performed at the end of each week. (**B** and **C**) Images of the cuff electrode (marked in red),

including the built-in sutures, connecting wires and head port. (**D**) An image of the cuff electrode implanted onto the sciatic nerve of the mouse and secured in place using the built-in sutures. The head port and connecting wires are passed through the subcutaneous layer and brought out through a gap made in the scalp. (**E**) An image of the head port secured to the scalp using dental putty. (**F**) Electrochemical impedance spectroscopy conducted using a three-electrode configuration – silver/silver chloride (Ag/AgCl) as a reference and platinum (Pt) as the counter electrode – and phosphate buffered saline (PBS) as the medium. A scan rate of 50 mV/s and frequencies from 1 Hz to 100 kHz were used. (**G**) Using the same testing setup, the charge storage capacity (CSC) was evaluated by cyclic voltametry (CV) using a scan rate of 50 mV/s. The CSC of the electrode was 2.28 mC/cm^2.

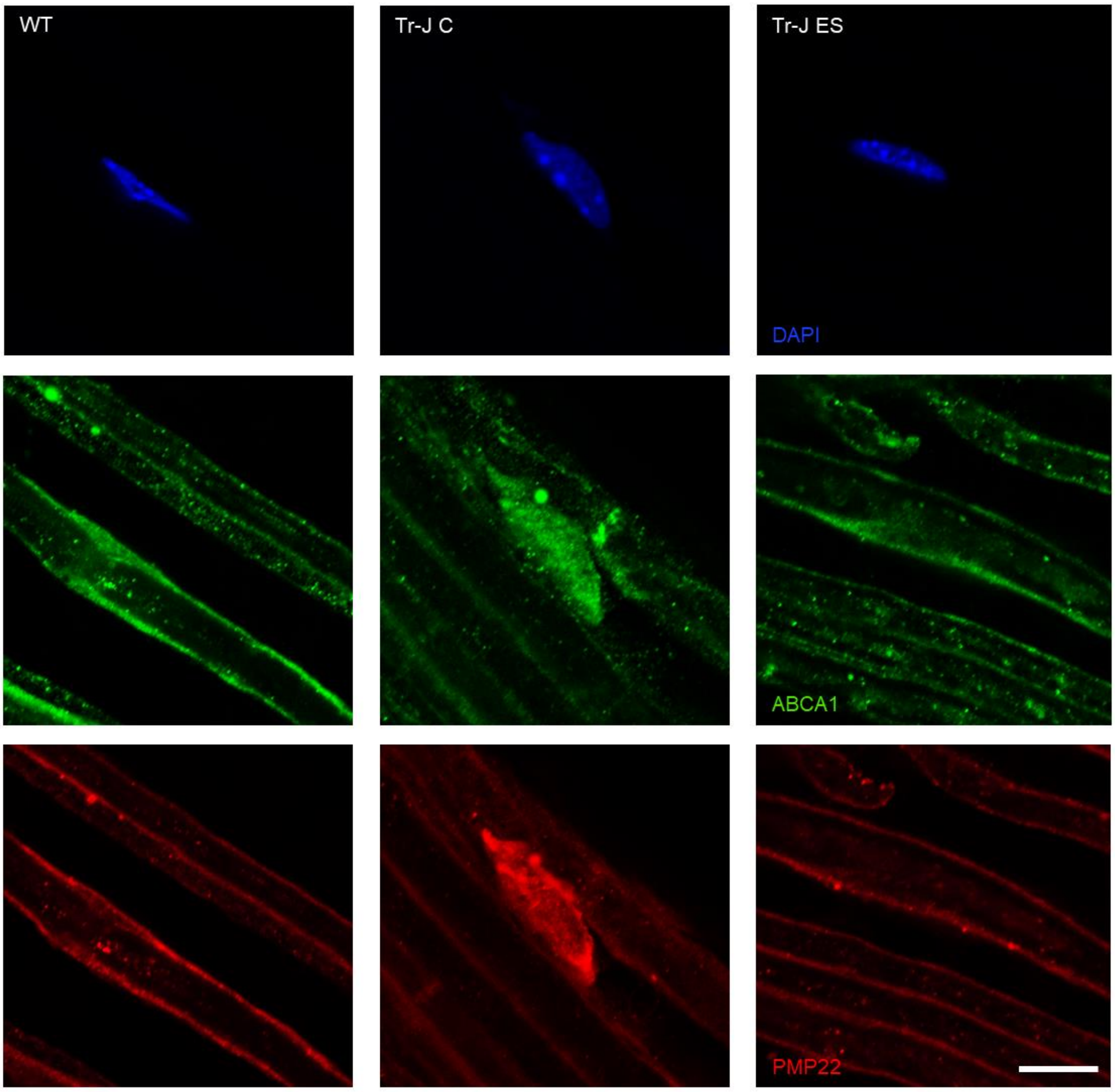

Figure S6. PMP22 and ABCA1 distribution in longitudinal sections of sciatic nerves. While ABCA1 and PMP22 are colocalized consistently along the membrane in WT mice, they tend to form perinuclear aggregates in Trembler-J (Tr-J) mice. After ES treatment, this distribution abnormality is considerably improved. Scale bar, 10 µm.

Table S1. Composition of growth, differentiation and myelination media.

	Growth	Differentiation	Myelination
**Base medium**	Neurobasal	DMEM/F-12	MEM Eagle
**Horse serum**	-	-	5%
**NGF**	50 ng/mL	50 ng/mL	50 ng/mL
**Ascorbic acid**	-	-	50 µg/mL
**N2 supplement**	-	1×	1×
**BPE**	-	-	20 µg/mL
**Forskolin**	-	-	0.5 µM
**Ab/Am**	1×	1×	1×
**L-Glutamine**	2 mM	2 mM	2 mM
**D-Glucose**	-	-	2.5 g/L
**B-27**	2%	-	-

MEM Eagle: Minimum Essential Medium Eagle (Sigma-Aldrich; catalogue number M4655). DMEM/F-12: Dulbecco's Modified Eagle Medium: Nutrient Mixture F-12 (Gibco; catalogue number 21331020). N-2 Supplement 100× (Gibco; catalogue number 17502048). L-Glutamine (Gibco; catalogue number 25030081). Ab/Am: Antibiotic-Antimycotic 100X (Gibco; catalogue number 15240062). BPE: Bovine Pituitary Extract (Gibco; catalogue number 13028014). D-Glucose (Sigma-Aldrich; catalogue number G8769). Forskolin (Cayman Chemical; catalogue number 11018). Nerve Growth Factor (Alomone Labs; catalogue number N-100). Horse Serum (Gibco; catalogue number 26050088). Ascorbic acid (Sigma Aldrich; catalogue number A92902).

Table S2. Sequences of primers used in this study.

Full name	Acronym	Forward sequence	Reverse sequence
**Glyceraldehyde 3-phosphate dehydrogenase**	GAPDH	TGCCCCCATGTTTGTGATG	TGTGGTCATGAGCCCTTCC
**Myelin basic protein**	MBP	ATCCCAAGGGAAGGGGAGAG	TCTGCCTCCGTAGCCAAATC
**c-Jun**	c-Jun	TGAGTGACCGCGACTTTTCA	GCATCGTCGTAGAAGGTCGT
**Activating transcription factor 4**	ATF4	GCCGGTTTAAGTTGTGTGCT	CTGGATTCGAGGAATGTGCT
**Activating transcription factor 6**	ATF6	GATGCAGCACATGAGGCTTA	CAGGAACGTGCTGAGTTGAA
**Binding immunoglobulin protein**	BiP	TGCAGCAGGACATCAAGTTC	TACGCCTCAGCAGTCTCCTT
**C/EBP homologous protein**	CHOP	CGGAACCTGAGGAGAGAGTG	CGTTTCCTGGGGATGAGATA
**3-hydroxy-3-methylglutaryl-coenzyme A reductase**	HMGCR	TGAGATCCGGAGGATCCAAG	GGGCACTCATAATTCCAGCC
**3-hydroxy-3-methylglutaryl-coenzyme A synthase**	HMGCS	TGATCCCCTTTGGTGGCTGAA	AGCTGTGTGAAGGACAGAGAAC
**Sterol regulatory element-binding protein 2**	SREBP2	GACCTCACGGGGGACTCTG	ACAAACTGTAGCATCTCGTCG
**Squalene synthase**	SQS	ACCCAAGATGGACCAGGACT	TGTATCCAGGGCTCGGAGAA
